# Child height, health and human capital: Evidence using genetic markers

**DOI:** 10.1016/j.euroecorev.2012.09.009

**Published:** 2013-01

**Authors:** Stephanie von Hinke Kessler Scholder, George Davey Smith, Debbie A. Lawlor, Carol Propper, Frank Windmeijer

**Affiliations:** a Department of Economics and Related Studies, University of York, Heslington, York YO10 5DD, UK; b CMPO, University of Bristol, 2 Priory Road, Bristol BS8 1TX, UK; c MRC Centre for Causal Analyses in Translational Epidemiology (CAiTE), School of Social and Community Medicine, University of Bristol, Oakfield House, Oakfield Grove, Bristol BS8 2BN, UK; d CMPO and Department of Economics, University of Bristol, 2 Priory Road, Bristol BS8 1TX, UK; e Imperial College London, South Kensington Campus, London SW7 2AZ, UK; f Centre for Microdata, Methods and Practice, UK

**Keywords:** Child height, Human capital, Instrumental variables, Mendelian randomization, Genetic variants

## Abstract

Height has long been recognized as being associated with better outcomes: the question is whether this association is causal. We use children's genetic variants as instrumental variables to deal with possible unobserved confounders and examine the effect of child/adolescent height on a wide range of outcomes: academic performance, IQ, self-esteem, depression symptoms and behavioral problems. OLS findings show that taller children have higher IQ, perform better in school, and are less likely to have behavioral problems. The IV results differ: taller girls (but not boys) have better cognitive performance and, in contrast to the OLS, greater height appears to increase behavioral problems.

## Highlights

► We examine whether height in children causally affects a wide range of outcomes. ► We use children's genetic variants as instrumental variables for child height. ► The results suggest that height is an important factor in human capital accumulation. ► We show that being tall may not only confer advantage but also disadvantage.

## 1 Introduction

The association between height and wealth has been noted in the academic literature for many decades. As early as the 17th Century, Guarinoni – one of the founders of preventive medicine – pointed to the difference in growth rates between the rich in towns and the poor in the countryside (Tanner, 1982). More recent studies find height to be positively related to education (Magnusson et al., 2006) and income (Persico et al., 2004). The advantages associated with greater height have also been reported for children. For example, Case and Paxson (2008) find that taller children perform better in school tests compared to shorter children and suggest that the relationship between childhood height and income and education in adulthood is due to height being associated with greater intelligence.

One problem in estimating the relationship between height and outcomes is that the relationship may not be causal. Height is influenced by a wide range of environmental factors experienced in childhood which may be the determinants of the outcomes, rather than height per se, for example, unobserved family wealth or differences in children's nutrition. To the extent that some of these unobserved differences are family specific, one approach is to identify the causal impact from twin or sibling differences in height and outcomes. Case and Paxson (2010) use this approach, exploiting differences between siblings. They conclude that taller children perform better in school, progress faster through school and consider themselves more scholastically competent than their shorter siblings.

However, accounting for fixed unobserved family effects using twin (or sibling) differences does not necessarily eliminate the inconsistency of the conventional cross-sectional estimator and can even aggravate it (Griliches, 1979, Bound and Solon, 1999). The intuition is that taking twin or sibling differences filters out some, but not all, endogenous variation but also filters out exogenous variation. If the endogenous variation comprises as large a proportion of the remaining within-sibling variation as it does of the between-sibling variation, the parameters using within-sibling estimation are as vulnerable to endogeneity bias as that found in between-sibling estimation. For example, some potentially endogenous variation that may remain in a within-sibling estimation are unobserved differences between siblings in nutrition and physical activity, both of which affect growth and final attained height. To argue that the within-sibling estimator is more consistent than the between-sibling estimator, this endogenous variation *as a share of the total variation* should be less in the within than the between-sibling estimation. There is no reason to be confident that this is the case, as the within-sibling analysis also removes exogenous variation, which – together with the endogenous variation – determines the inconsistency (Bound and Solon, 1999).

This paper therefore takes a different approach to estimate the causal effect of child height on children's cognitive and non-cognitive outcomes. Our approach is also called Mendelian randomization, which refers to the use of genetic variants as instrumental variables (IV) to examine the causal effect of an exposure (here height) on outcomes. It exploits the random assignment of an individual's genotype at conception (Davey Smith and Ebrahim, 2003) to enable genetic variants to instrument for a particular phenotype (the trait that the genetic variants is related to, e.g. height).^1^ At conception, genes are randomly allocated from parents to offspring. Whilst this random allocation is at a family trio level, at a population level it has been demonstrated that genetic variants are largely unrelated to the many socioeconomic and behavioral characteristics that are closely linked with each other and that confound conventional observational studies (Bhatti et al., 2005, Davey Smith et al., 2008, Kivimäki et al., 2008, Lawlor et al., 2008). Furthermore, since genetic variation is determined at conception, it cannot be affected by later outcomes. Hence, in addition to dealing with fixed characteristics that affect both height and the outcome, Mendelian randomization can also deal with *time-varying* characteristics that affect height and outcomes. Therefore, under certain assumptions that we discuss below, genetic variants will allow us to isolate the causal effect of child height on the outcome of interest.

This paper is the first to exploit genetic variants for height in an attempt to estimate the causal effect of height on cognitive and non-cognitive outcomes for children. We begin therefore by outlining the conditions needed to use genetic variants as instruments. To examine and indirectly test the validity of the IV approach in our context, we show first that the genetic variants are uncorrelated with a large set of family background variables which may confound the relationship between height and outcomes. We then discuss biological pathways of our genetic variants, and run two ‘falsification checks’. First, we examine the effect of height on an outcome for which we have clear theoretical reasoning that there should *not* be an effect (maternal education). And second, we investigate the effect of height on an outcome for which we have strong beliefs that there *should* be an effect (body weight). Finding no evidence against the validity of the instruments, we then use the genetic variants as instruments to examine the relationship between height and an extensive set of cognitive, mental health and behavioral outcomes. In so doing, we add to the range of outcomes examined in the previous literature. In addition to children's academic attainment, scholastic competence and self-worth studied by Case and Paxson (2010), we investigate the effects of height on IQ, symptoms of depression and behavioral problems, including hyperactivity, emotional, conduct and peer problems. Note here, that our IQ measure is an index of general intellectual functioning, which is shaped by both inherited and acquired attributes, including any family and environmental influences. In other words, it does not simply measure ‘innate’ ability.

We use data from a cohort of UK children currently in their late teens (the ALSPAC survey, described below). The OLS results show that taller children perform better in school tests, have higher IQ, and are less likely to have emotional and peer problems, though these relationships differ slightly by gender. Tall girls have higher depression scores, but we find no evidence of differences in self-esteem for children of different heights. The IV results suggest there is a causal relationship between height and cognitive functioning, though only robustly for girls. In contrast to Case and Paxson (2010), we find no evidence that height explains variation in scholastic self-esteem, global self-worth or depression. Further, we find evidence that height confers disadvantage rather than advantage as it *increases* hyperactive behavior (girls), emotional and peer problems (boys). These findings are robust to a set of instrument specification and robustness checks. We discuss the results, relating back to the assumptions made in Mendelian randomization, and speculate about possible reasons for these findings.

The next section begins by examining the possible mechanisms through which height may be related to the outcomes of interest. In Section 3, we set out our methodology and Section 4 describes the data. The results are presented in Section 5 and Section 6 concludes.

## 2 Mechanisms

We examine a large set of outcomes: academic attainment, IQ, self-esteem, depression symptoms, and behavioral problems. There are two ways in which height may be related to these outcomes. First, being tall could cause differences in the outcome of interest. We define ‘causal’ however, not necessarily as height per se affecting the outcome, but as height triggering social reactions that in turn affect the outcome. Hence, we hypothesize the effect of height to run via different pathways, which we discuss below. Second, instead of there being a causal relationship, the association between height and the outcome of interest may be driven by other unobserved factors that affect both.^2^

Several pathways through which height can causally affect outcomes are discussed in the literature, including taller people being more competitive (Fessler et al., 2010), enjoying social dominance (Hensley, 1993) and having higher self-esteem (Judge and Cable, 2004). In a field experiment asking participants to choose between a competitive and non-competitive payment scheme, Fessler et al. (2010) find that, controlling for gender, the tallest quartile are one-and-a-half times more likely to choose the competitive scheme compared to the shortest quartile. The (sociological and psychological) literature posits several theories as to why (physical) characteristics may affect behavior or achievement. First, the possession of certain characteristics (like being tall) can trigger expectations from others (like peers or teachers). These expectations may influence their behavior towards the ‘possessor’, which in turn affects the possessor's behavior, often confirming the expectations. This self-fulfilling prophecy is also referred to as the ‘expectancy effect’ (see Darley and Fazio, 1980). For example, some evidence suggests that taller people are perceived as more attractive (Macintyre and West, 1991). Attractiveness can in turn influence the behavior and assessment of teachers (Clifford and Walster, 1973) or potential employers (Dipboye et al., 1975), causing taller people to behave and perform differently.

Second, short children are believed to have negative social experiences, including bullying, less social acceptance, and fewer friends (Sandberg and Voss, 2002, Voss and Mulligan, 2000),^3^ though it is worth noting that tallness in girls has also been shown to have similar negative psychological effects (Pyett et al., 2005, Binder et al., 1997). Having problematic social relationships can in turn affect self-esteem, social adjustment, behavior, and scholastic performance (Morison and Masten, 1991, Parker and Asher, 1987, Wentzel, 2009). Related to this is the question of whether parents compensate or reinforce children's endowments, the evidence of which is mixed, see e.g. Griliches, (1979) and Behrman et al. (1994). The former may mean that parents spend relatively more time with a small compared to a tall child, to compensate for the potential negative experiences related to short stature. As the child develops through childhood, this additional attention and support can in turn increase their cognitive skills, or reduce their behavioral problems.

Another strand of the literature suggests that individuals (peers, parents, teachers as well as medical personnel) treat children at a ‘size-appropriate’ rather than ‘age-appropriate’ level: tall children are generally perceived to be (and treated as) older, whereas smaller children are treated as younger (Jones and Bayley, 1950, Rotnem et al., 1977, Underwood, 1991, Sandberg et al., 2004). Adults in turn may have different expectations depending on children's heights (Skuse et al., 1994), which can subsequently affect children's behavior. Children who ‘look young’ according to their peers are perceived to be less (physically and verbally) aggressive and more emotional and passive (Sandberg et al., 2004). In addition, the literature has found taller children to have more behavioral problems, such as aggression or violent behavior. Raine et al. (1998) find that height in 3-year-old children is associated with increased aggressiveness at age 11, and Farrington (1989) find that height at age 8–10 years is associated with violence at age 16–18 years. They argue that their early life may have taught them that it is an effective strategy in winning social conflicts, reinforcing this behavior. In contrast, smaller and physically weaker children lack the physical capacity to execute this behavior (Raine et al., 1998).

As opposed to a causal effect, there may be other factors that relate to both height and the outcome of interest and that drive the associations. One set of candidates is the pre- and postnatal environment. Regarding the latter, the fastest growth in children occurs up to age 2. There is evidence of links between early (post-natal) nutrition and child height, and between nutrition and cognitive and social development. For example, iron-deficiency in infants and children is associated with poorer cognitive, motor and socio-emotional function (see e.g. Lozoff et al., 2006). In addition, some studies report that iron supplementation positively affects height (Angeles et al., 1993). But although early nutrition is a possible candidate, several studies have shown that even under conditions of severe malnutrition (prenatal, such as fetuses subjected to war-time famine and postnatal, such as starvation in the early years of life) complete equality in height with siblings or peers is attained before puberty (Tanner, 1978).

In terms of the pre-natal diet, there is evidence that nutrition *in utero* plays an important role in child development. But nutriments which help some developmental aspects, may hurt others. For example, omega 3 fatty acids in fish and seafood consumption are crucial for brain development and have been associated with decreased hostility and aggression (Benton, 2007), but are also the primary source of (non-occupational) mercury exposure (Oken and Bellinger, 2008). Several studies have shown prenatal methylmercury exposure to be associated with decreased IQ and test scores (Axelrad et al., 2007, Cohen et al., 2005). Likewise, some studies find that maternal alcohol consumption and smoking during pregnancy negatively affect birth weight and child growth (Mills et al., 1984, Gilman et al., 2008). Lower birth weights in turn are associated with poorer cognitive performance (Richards et al., 2002, Ericson and Kallen, 1998) and behavioral development (Elgen et al., 2002), though the literature suggests that this relationship is driven by family background characteristics rather than a specific intrauterine effect (Yang et al., 2008). There is mixed evidence on the effects of maternal smoking and alcohol consumption during pregnancy on child outcomes, with some arguing it lowers outcomes and others finding no effect (see e.g. Olds et al., 1994, Gilman et al., 2008, Kafouri et al., 2009, Davey Smith, 2008, Nilsson, 2008, Russell, 1991).

Other potential confounders include genetic causes of both height and the outcome of interest. This may be especially important in this context, as both height and (for example) cognition are likely be influenced by a large number of genes, each with very small effects. Indeed, some literature suggests that part of the height–intelligence association is driven by a genetic component (Sundet et al., 2005), though others find no evidence of this. For instance, comparing first and second born biological brothers in Sweden, Magnusson et al. (2006) find that the taller brother is significantly more likely to attend higher education. However, the height effect estimated between brothers is almost identical to that across all men, suggesting that the correlation between height and intelligence is not driven solely by genetic or environmental factors common to brothers.

This discussion suggests that a potential bias can go in either direction. If a well-balanced diet or the family's socio-economic position positively affects height, but also leads to fewer behavioral problems, the OLS is likely to under-estimate the true effect of height on behavioral problems. If, however, this same diet leads to better educational outcomes, OLS is likely to over-estimate the true effect on education. However, if certain dietary components lead to decreased cognitive functioning, the OLS may under-estimate the true effect on educational outcomes and IQ. Under the assumptions we discuss in detail below, the use of the child's genetic markers as instrumental variables will shed more light on these issues and will allow us to estimate the causal effect of child height.

## 3 Methodology

### 3.1 The potential outcomes framework

We examine the impact of child height on three sets of outcomes: (1) cognitive skills, (2) mental health, and (3) behavioral problems. We discuss the outcomes in more detail below. As both height and outcomes differ by gender, we estimate all models separately for boys and girls. We model the relationship between height and outcomes using the potential outcomes framework, building on the work by Imbens and Angrist (1994) and Angrist et al. (1996), which has been of great importance in linking the econometric IV literature to the potential outcomes framework.

Let *C*, *H* and *Z* denote random variables representing, respectively, the outcome of interest, child height and the genetic variant as IV. For simplicity, we initially discuss the case of a binary instrument, though we consider the case of multi-valued instruments below. *Z*_*i*_=1 indicates that individual *i* carries the genetic variant, *Z*_*i*_=0 implies that individual *i* does not carry the genetic variant.

Let *H*_*i*_(*z*) be the potential height for individual *i* when the instrument is set equal to *z*. Equivalently, let *C*_*i*_(*h*,*z*) be the potential outcome for individual *i* that would be obtained if height, the treatment variable, was set to *h* and the instrument set to *z*. We refer to *H*_*i*_(*z*) and *C*_*i*_(*h*,*z*) as the potential treatments and potential outcomes respectively.

The individual treatment effect, or causal effect, is *C*_*i*_(*h*′,*z*)−*C*_*i*_(*h*,*z*), where *h* is some baseline value. Under the exclusion restriction discussed below, we can write *C*_*i*_(*h*′,*z*)=*C*_*i*_(*h*′). The causal estimand of interest can therefore be written as

(1) $$E[C_{i}(h\prime )-C_{i}(h)].$$

We follow Angrist et al. (2000), who specify the conditions under which the simple IV estimator identifies a weighted average of the derivative function of the non-linear causal response function. We discuss these assumptions in turn.

Assumption 1 (Independence)

$$Z_{i}⊥{\left\{C_{i}(h,z),H(z)\right\}}_{h,z}$$

Independence implies that the instrument is independent of the potential outcome and the potential height, for all values of *h* and *z*. In other words, the instrument is as good as randomly assigned.

Assumption 2 (Exclusion)

$$C_{i}(h,1)=C_{i}(h,0)\;\mathrm{for}\;\mathrm{all}\;h.$$

Exclusion implies that the potential outcomes, at any height *h*, are unchanged by the presence or absence of the genetic variant. In other words, the only way through which the instrument affects the potential outcome is via *H*.

Assumption 3 (Nonzero effect of instrument on height)

$$E[H_{i}(1)-H_{i}(0)]\neq 0$$

This implies that expected potential height is affected by the genetic variant and therefore, that the instrument has an effect on treatment.

Assumption 4 (Monotonicity)

$$P[H_{i}(1)\geq H_{i}(0)]=1\;\mathrm{for}\;\mathrm{all}\;(\mathrm{or}\;\mathrm{vice}\;\mathrm{versa})$$

This means that the potential height for individual *i* with the genetic variant is at least as high as the potential height for the same individual without the genetic variant.

Specifying heterogeneous responses, the potential outcome for individual *i* can be written as a general function of *h*, say *C*_*i*_(*h*)≡*g*_*i*_(*h*). Under the assumptions above, the instrumental variables estimand, defined as the ratio of the difference in average outcomes at two values of the instrument to the difference in average treatment at the same two values of the instrument, can be written as

(2) $$\frac{E[C_{i}|Z_{i}=1]-E[C_{i}|Z_{i}=0]}{E[H_{i}|Z_{i}=1]-E[H_{i}|Z_{i}=0]}=\frac{\int^{\;}E[g_{i}^{'}(q)|H_{i}(0)<q<H_{i}(1)]P\{H_{i}(0)<q<H_{i}(1)\}dq}{\int^{\;}P\{H_{i}(0)<q<H_{i}(1)\}dq},$$

where $g_{i}^{'}(q)$ is the derivative of *g*_*i*_(*h*) w.r.t. *h* evaluated at *q*. Therefore, the IV estimator is a weighted average of the derivative function (Angrist et al., 2000, Angrist and Pischke, 2009).

Although the above discussion uses a binary instrumental variable, we observe a multi-valued instrument. In the case of such discrete instruments, the IV estimate is a weighted average of the average causal derivatives calculated at each value of the instrument, where the weights are determined by the strength of the instrument on the treatment. Hence, the IV estimate is a weighted average of the derivative function at the different values of the instrumental variable (Angrist et al., 2000).

### 3.2 The genetic variants

We use a set of nine genetic variants (single-nucleotide polymorphisms: SNPs (see glossary, Table 1, and the Appendix)) that have all been robustly associated with height among individuals of European ancestry. The nine variants we use are SNPs in the following genes: *HMGA2* (rs1042725), *ZBTB38* (rs6440003), *GDF5* (rs6060373), *LOC387103* (rs4549631), *EFEMP1* (rs3791675), *SCMH1* (rs6686842), *ADAMTSL3* (rs10906982), *DYM* (rs8099594) and *C6orf106* (rs2814993), where the rs number is a unique SNP identifier.^4^

**Table 1:** A glossary of some genetic terms.

Term	Definition
Alleles	One of two or more versions of a specific location on the DNA sequence. An individual has two alleles, one from each parent
Base	Also called nucleotide. Bases are the ‘building blocks’ of DNA. DNA consists of four bases: adenine (A), cytosine (C), guanine (G) and thymine (T). It is the sequence of these four bases that encodes information
Chromosome	A continuous piece of DNA that carries a collection of genes. Every cell in the human body contains 46 chromosomes
DNA	Deoxyribonucleic acid (DNA) contains the genetic instructions used in the development and functioning of all living organisms. The DNA segments that carry the genetic information are called genes. The double-helix structure joins two strands of DNA, where the base A binds with T, and G binds with C
Gene	A section on the chromosome that comprises a stretch of DNA
Genotype	The specific set of two alleles inherited at a particular location on the DNA sequence. If the alleles are the same, the genotype is homozygous. If different, it is heterozygous
Homozygous	When the two alleles at a particular locus are the same
Heterozygous	When the two alleles at a particular locus are different
Heritability	The proportion of the total variance that is explained by genetic factors. It is most commonly calculated from twin studies by comparing intra-pair correlations for the characteristic in monozygotic (MZ) with intra-pair correlation in dizygotic (DZ) twins. The heritability is of a characteristic is calculated as twice the difference between MZ and DZ intra-pair correlations (*h*^2^=2⁎(*r*_*MZ*_−*r*_*DZ*_))
Linkage disequilibrium (LD)	The correlation between alleles at different loci within the population that occurs due to the co-inheritance of alleles. Alleles that are in LD are not independent of another. The extent of LD is a function of the distance between the alleles on the chromosome
Phenotype	An organism's observable characteristic or trait, such as its biochemical or physiological properties. Phenotypes result from the expression of genes as well as the influence of environmental factors and the interaction between the two
Pleiotropy	The potential for variants to have more than one phenotypic effect. If a SNP is pleiotropic, it influences multiple phenotypes
Polymorphism	Locations where DNA varies between individuals
Population stratification	The presence of a systematic difference in allele frequencies between subpopulations within a population. The most common example is population stratification due to ethnicity
Single-nucleotide polymorphism (SNP)	A genetic variation in which a single base/nucleotide on the DNA is altered, e.g. the nucleotide T is changed to A

Mendelian randomization is valid assuming that, at the population level, the genetic variants are unrelated to the type of unmeasured lifestyle and socio-economic confounders that tend to distort interpretations of observational studies. The theory of random allocation of genetic variants and the empirical evidence on this suggest this is the case (Bhatti et al., 2005, Davey Smith et al., 2008, Kivimäki et al., 2008, Lawlor et al., 2008; see also Fisher, 1952, Box, 2010, Bodmer, 2010). We discuss the assumptions in turn, relating this to our research question.^5^

#### 3.2.1 Assumption 1: Independence

One way to indirectly test Assumption 1 is by exploring whether the distribution of individual or family-level characteristics that are available in the data is the same in different groups defined by the value of the instrument. In Section 4.4, we examine the relationship between the genetic variants and a large set of child and family background characteristics. The idea is that, if the instrumental variable is indeed randomized, there should be no systematic variation in the covariates by genotype. This raises the question however, about *which* covariates to test for, as any characteristic is, in principle, a post-treatment variable with respect to the instrument. Hence, any systematic variation in these indirect tests does not *necessarily* indicate a violation of independence (or exclusion). It may be, for example, that the instrument is picking up additional causal effects of the same risk factor, or that it is picking up reverse causation from the outcome to a different covariate.

One way through which the independence assumption can be violated is *population stratification*. This refers to a situation in which there is a systematic relationship between the allele frequency and the outcome in different population subgroups (see Table 1 and the Appendix for a definition of some genetic terms). For example, allele frequencies can vary across ethnic groups. If these groups also have systematically different educational outcomes that are not due to a genetic make-up, this could lead to an association between the two at the population level without an actual causal relationship, violating the independence Assumption 1. In other words, despite the fact that genotypes are randomly allocated and with that satisfy Independence, any population stratification can violate this assumption. This can be dealt with however, by examining the question of interest *within* ethnic groups, separately analyzing the different sub-populations, and/or adjusting for principal components from genome wide data that function as ancestry markers, relying on the *conditional* independence assumption. Population stratification is unlikely to affect our estimation, as our cohort is recruited from a specific geographically defined region, and fewer than 3% of the mothers reported that either they or their partner were from an ethnicity other than White European. With this small number of participants removed, a principal components analysis using genome-wide data in the cohort suggests that it consists of one population.

#### 3.2.2 Assumption 2: Exclusion

There are various situations that can violate the exclusion restriction. First, as individuals inherit their genes from their parents, it may be important to consider whether parents’ behaviors are affected by their genotype (and hence are related to their offspring's genotype). In the presence of strong ‘dynastic effects’, genetic instruments may be invalid if they are related to parental behaviors that in turn affect the outcome of interest (Fletcher, 2011). For example, parents who carry ‘tall’ alleles may be treated differently because of their taller stature. If this affects their preferences for their child's education, Assumption 2 may be violated. The extent of this potential violation however, will depend on the effect sizes of the variants. In our case, the genetic variants increase the average height by a relatively modest amount, which is unlikely to lead to strong (parental) responses.

Second, if the variants have multiple functions (also known as pleiotropy), Assumption 2 could be violated. This would occur for example, if – over and above the association with height – the variant has a direct effect on our outcome of interest (such as cognition or self-esteem), violating the exclusion restriction. Similarly, if a variant is co-inherited with another genetic variant (known as being in linkage disequilibrium (LD)), violation of Assumption 2 depends on the effect of the co-inherited variant on the outcome of interest. The current evidence suggests that some height variants may indeed be pleiotropic or in LD (i.e. co-inherited) with other variants. For example, individuals with higher levels of GDF5 on average have both increased bone and cartilage growth (Sanna et al., 2008). However, there is currently no evidence that the variants used here additionally directly affect (or are in LD with variants that directly affect) our outcomes of interest or determinants thereof.

We investigate the potential violation of the IV assumptions in a number of ways. First, we search the literature to identify evidence on the biological pathways of our variants, which may shed more light on the mechanisms through which they affect height. Medical and theoretical evidence that suggest that the SNPs only affect the outcome *through* their effect on height would in turn mitigate concerns about the exclusion restriction. Although the biological pathways are not known for all variants, Allen et al. (2010) show that a substantial number of the 180 SNPs they study, including some used here, are involved in growth-related processes.^6^

Despite the absence of evidence of our SNPs directly affecting (determinants of) the outcomes of interest, and despite the biological pathways pointing to skeletal development and cell growth, we cannot guarantee that Assumption 2 holds. For instance, it is possible that some variants’ pleiotropic effects (i.e. any additional effects independent from those on height) have simply not yet been identified. The 180 SNPs that have so far been identified explain 10% of the total variation in height. Hundreds, maybe thousands more effects are still lost in the genome (McEvoy and Visscher, 2009). Hence, it is possible for one (or more) of the nine instruments used here to be pleiotropic or in LD with a variant that directly affects our outcome. Based on the best available evidence however, we assume this is not the case and that Assumption 2 holds.^7^ We reiterate though, that – *similar to any other IV approach* – this remains an assumption, as we cannot test for this directly. In other words, its validity will never be known with complete certainty and can only be examined indirectly or falsified by the data.

When data are available on a large number of variants affecting the risk factor of interest, genetic confounding through pleiotropy or LD can be examined in more detail. More specifically, if multiple IV models – each using different independent combinations of these variants – predict a similar causal effect, this is very unlikely to be due to some common pleiotropy or LD across the different sets of variants, assuming that the different variants are located on different chromosomes and affect the trait via different pathways (Davey Smith, 2011, Palmer et al., 2011). Hence, if the different IV specifications display consistency, it provides some evidence against genetic confounding. One would ideally have a large number of variants available to thoroughly test for this, allowing for many different combinations of instrument sets without having to deal with weak instruments. Although the genetic data available to us is more limited, we explore this concept and investigate this further in Section 5.3.

#### 3.2.3 Assumption 3: Nonzero effect of instrument on height

The prior knowledge on the effects of the variants, our use of a comparable sample of individuals of European ancestry, and the fact that these associations have been replicated in different independent samples, justify the use of these variants and their compliance with Assumption 3. However, as gene–environment interactions in different samples can violate this Assumption (see e.g. von Hinke Kessler Scholder et al., 2011a), Section 5.2 examines the strength of the instrument in our sample, using the standard statistical tests. Although the relationships between the SNPs and height are robust, their phenotypic effects (the actual effects on height) are small. In our analysis, we therefore combine the different SNPs into a count of the number of ‘tall’ alleles carried by each child to get around the problem of low power. We create a count of the total number of height-increasing alleles for each child and use this as the instrumental variable for height (see Section 4.4).

#### 3.2.4 Assumption 4: Monotonicity

Given random allocation of genetic variants and the fact that individuals do not know their genotypes, we assume that an individual who carries a ‘tall’ allele is at least as tall as the same individual, had she not carried the ‘tall’ allele, thus satisfying the monotonicity Assumption 4. As this relies on knowing each individual's counterfactual, this remains an assumption. The literature only shows that, at a group or population level, those who possess the genetic variant are taller than those who do not. The assumption could, for example, be violated in the presence of gene–environment interactions, though we are not aware of any evidence of this for the SNPs used here.

## 4 Data

We use data from a cohort of children born in the Avon area of England. Avon has approximately 1 million inhabitants, including 0.5 million in its main city, Bristol. Women eligible for enrollment in the population-based Avon Longitudinal Study of Parents and Children (ALSPAC) had an expected delivery date between 1 April 1991 and 31 December 1992. Approximately 85% of these mothers enrolled, leading to about 14,000 pregnancies. The Avon area is broadly representative of the UK, though mothers were slightly more affluent compared to the general population (Golding et al., 2001; see www.bris.ac.uk/alspac for a more detailed description of the sample, its enrollment, and response rates). Note that ALSPAC is a cohort; there is no systematic data collection on siblings.

Detailed information on the children and their families has been collected from a variety of sources, including self-completed questionnaires, data extraction from medical and educational records, in-depth interviews, and clinical assessments and so our data contain a large range of child health and development, family background, family inputs and school measures.

A total of 12,620 children survived past the age of 1 and returned at least one questionnaire. Of these, 642 were excluded because either their mother or father is of non-white ethnic origin, leaving 11,978 potential participants. Our sample selection process is as follows. First, we select those children for whom we observe all nine genotypes, leaving us with approximately 7100 children. Second, we drop children for whom we do not observe their height. Children were invited to attend specially designed clinics, where their anthropometric measures were recorded. As not all children attended these clinics, our sample sizes reduce to between 4594 (age 8) and 3867 (age 13). Finally, we restrict the sample to those children for whom we observe the outcome of interest, leading to a final sample size of around 3900 at age 8 and 3300 at age 13. We deal with missing values on other covariates by using multivariate imputation (Royston, 2004).

### 4.1 Outcome measures

We examine three sets of outcomes. First, we observe two measures of cognitive function. These are the child's score on the nationally set Key Stage 3 (KS3) exam (taken by all 14-year-olds educated in the state sector) and the child's IQ, measured as age 8.^8^ Both measures are objective and comparable across all children. Increasing scores indicate better performance. It is important to note that IQ does not only measure ‘innate’ ability. Instead, our measure of IQ (WISC-III) is an index of general intellectual functioning, which is shaped by both inherited and acquired attributes, including any family and environmental influences. For example, there is evidence of differences in IQ between children of different quality home environments and socio-economic position (see e.g. Molfese et al. (1997) and references therein).

Second, we examine three measures of mental health or self-esteem: depression symptoms, scholastic competence and global self-worth. The latter two are measured at age 8, using the Harter's Self-Perception Profile for Children (Harter, 1985), with increasing scores indicating higher self-esteem. The depression score is self-reported by the teenager at age 13 using the Moods and Feelings Questionnaire (Angold et al., 1995). Increasing scores indicate more depression symptoms.

Third, we examine the child's behavioral problems, as measured by the mother's report on the Strength and Difficulties Questionnaire (SDQ; Goodman, 1997) administered at age 13. SDQ has four sub-scores, which we examine separately (as is common in the literature). These are hyperactivity, emotional problems, conduct problems and peer problems. Increasing scores indicate increasing problems.

For comparability, all outcomes are standardized on the full sample of children for whom data is available, with mean 100, standard deviation 10.

### 4.2 Measures of child height and the genetic variants

We examine the effect of contemporaneous height on each outcome. Height is adjusted for the exact age in month at which it is measured and standardized to have mean 100, standard deviation 10. All measurements are taken by trained nurses. We instrument height with a set of SNPs that have been consistently shown to relate to stature. These are SNPs located in the following genes: *HMGA2*, *ZBTB38*, *GDF5*, *LOC387103*, *EFEMP1*, *SCMH1*, *ADAMTSL3*, *DYM* and *C6orf106*. All but two SNPs are located on different chromosomes (*LOC387103* and *C6orf106* are both on chromosome 6), with the correlation in our sample ranging from −0.029 to 0.026. Hence, each SNP has an independent effect on stature.

### 4.3 Covariates

The main reason for the inclusion of covariates in economics IV studies is that the *conditional* independence and exclusion restriction are more likely to be valid. A second reason for including covariates is that it may reduce the variability in the dependent variable, leading to more precise estimates. In Mendelian randomization studies, however, the theory and evidence on the random allocation of genetic variants suggests that we can rely on the *unconditional* independence and exclusion restriction. In fact, the inclusion of covariates may bias the estimates of interest. For example, if the instrumented risk factor (here: height) has multiple causal effects, or if the outcome of interest has a causal effect of its own on the covariates, adjusting for such post-treatment variables may lead to biased estimates of the causal effect of interest. Under the independence assumption and exclusion restriction, and in a situation where the instrumented risk factor and outcome do not (directly or indirectly) affect these covariates, the unadjusted and adjusted IV estimates should be similar, though the latter may be more precise. We present the main findings both with and without adjustment for covariates. These show similar results, providing at least suggestive evidence that the instruments satisfy independence and exclusion.

In the analysis that adjusts for covariates, we control for a rich set of child and family characteristics, including the child's birth weight and the number of older and younger siblings under 18 in the household. As the outcomes of interest may vary with within-year-age, we also account for the child's age (in months) at the time the outcome is measured. We control for the family's socio-economic position with various measures: log equivalized family income and its square, four binary variables for mother's and father's educational level, the mother's parents’ educational level, an indicator for whether the child is raised by the natural father, variables indicating the family's social class, and parents’ employment status when the child is 21 months. As a further measure, we include a measure of small (local) area deprivation, as measured at the child's birth.^9^

In addition to these generally observed controls, our data allow us to also account for several further measures of mother's health and behavior, which may be correlated with both child height and the outcome of interest. We use two binary variables which measure whether the mother smoked or drank alcohol in the first three months of pregnancy; an ordered indicator for the intensity of mother's breastfeeding (never, <1 month, 1–3 months and 3+ months); mother's age at birth (20–24, 25–29, 30–34, 35+); mother's ‘locus of control’, a psychological concept that describes whether individuals attribute successes and failures to internal or external causes (those with an external locus of control attribute success and failure to chance); two further measures of maternal mental health; and finally several measures of parental involvement or interest in the child's development.^10^

### 4.4 Descriptive statistics

Table 2 presents mean height (at age 8) for each of the SNPs, distinguishing between children who are homozygous for the height-increasing allele, heterozygous and homozygous for the height non-increasing allele (see the glossary in Table 1 and the Appendix for some of the genetic terms used here). These show that each of the individual SNPs explain little of the variation in child height. This would imply that the first stage regressions have low explanatory power, which could result in biased estimates. To avoid such problems of low power, we create a count of the total number of height-increasing alleles carried by each child (as in e.g. Weedon et al., 2008, Lettre et al., 2008). We use this in our main analysis as the instrument for child height. As shown by Pierce et al. (2010), combining genetic factors as such alleviates weak IV problems. However, they also show that such counts are mainly appropriate when variants have similar effects, but suboptimal otherwise, as the effect sizes will be mis-specified. Indeed, a simple count of the number of risk alleles imposes structure, setting the magnitude of the effects of all alleles to be equal. As an alternative, we therefore check the robustness of our results in Section 5.3, using a weighted allele score, where the weights are the gender-specific strengths of the association between the variant and individual height, as estimated by a large genome-wide association study of 183,727 individuals in 61 independent datasets (Allen et al., 2010). In this section, we also investigate the robustness of our results to the use of different combinations of different sets of instruments.

**Table 2:** Mean and standard deviation of height at age 8 (in cm) for each SNP.

Gene	SNP rs number	‘Tall’ allele	Homozygous for ‘non-tall’ allele		Heterozygous		Homozygous for ‘tall’ allele	
			Mean	Std. dev.	Mean	Std. dev.	Mean	Std. dev.
*HMGA2*	rs1042725	**C**/T	132.0	(5.47)	132.0	(5.59)	132.7	(5.71)
*ZBTB38*	rs6440003	**A**/G	131.8	(5.53)	132.3	(5.68)	132.4	(5.47)
*GDF5*	rs6060373	**C**/T	132.2	(5.58)	132.2	(5.59)	132.2	(5.71)
*LOC387103*/*C6orf173*	rs4549631	**C**/T	131.6	(5.45)	132.5	(5.68)	132.1	(5.54)
*EFEMP1*	rs3791675	A/**G**	131.0	(5.75)	131.9	(5.52)	132.4	(5.61)
*SCMH1*	rs6686842	**A**/G	131.9	(5.51)	132.2	(5.62)	132.4	(5.68)
*ADAMTSL3*	rs10906982	**A**/T	131.8	(5.53)	132.1	(5.64)	132.5	(5.57)
*DYM*	rs8099594	**T**/C	131.9	(5.72)	132.2	(5.51)	132.2	(5.65)
*HMGA1*/*C6orf106*	rs2814993	**T**/C	132.2	(5.70)	132.1	(5.34)	132.5	(5.19)

*Note*: The height-increasing allele is bold and underlined.

**Fig. 1:**
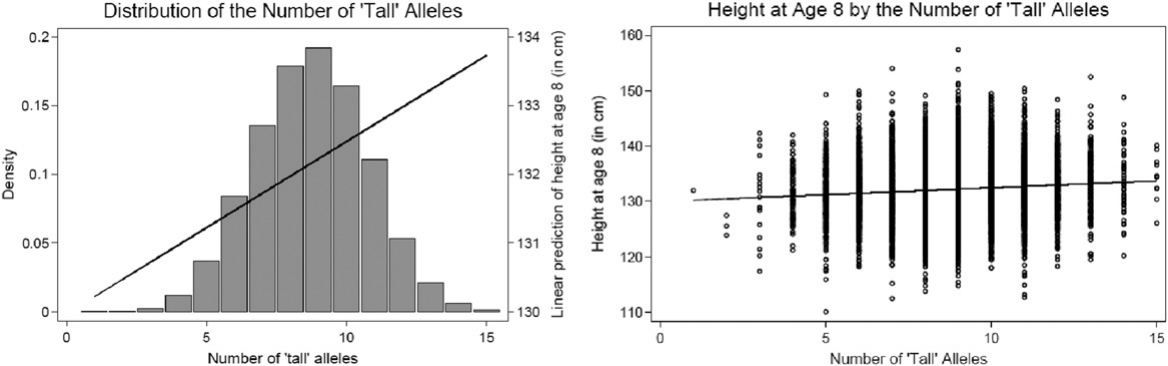
Histogram of children's height at age 8 by the number of height-increasing alleles.

The left panel of Fig. 1 presents a histogram of the number of ‘tall’ alleles carried by each child, showing a bell-shaped distribution. The linear prediction of height, obtained from a regression on the number of ‘tall’ alleles, is presented by the straight line. On average, each ‘tall’ allele increases the child's height at age 8 by 0.043 standard deviations (about 0.25 cm). There is, however, a considerable amount of unexplained variation in height (*R*^2^<1%), as shown in the right panel of Fig. 1, where the linear prediction is presented by the same straight line.

Columns 1 and 2 in Table 3 present the descriptive statistics (mean, standard deviation) of the variables discussed above. This shows an average height at age 8 of 132.2 cm and of 163.3 cm at age 13. In the analysis, we use standardized heights. Columns 3–5 show the raw association between this measure, the covariates and the number of ‘tall’ alleles, obtained from a regression of standardized height or each covariate on the number of height-increasing alleles. The top two rows of these columns present the relationship between child height and the instrument, showing a strong relationship for height at both ages. On average, each ‘tall’ allele is associated with a 0.043–0.047 standard deviation increase in child height (recall from above that height is distributed with mean 100, standard deviation 10). The rest of columns (3–5) show no clear patterns or (with three exceptions) statistically significant associations in the relationship between the contextual variables and the number of height-increasing alleles. Using a two-sided binomial probability test at the 5% level, a comparison of the *observed* versus *expected* number of significant correlations suggests that the genetic variants show no greater association with the child and family background characteristics than what would be expected by chance (*p=*0.15). Failing to reject the null, however, does not necessarily imply it is true. In other words, it does not guarantee that the instrument is orthogonal to any potential confounders, as it may be that the association is too small to detect with our sample size, or that we simply do not observe the relevant confounders. Nevertheless, it provides suggestive evidence that the instruments support Assumption 1, Assumption 2.^11^

**Table 3:** Descriptive statistics of height and the covariates: Columns 1 and 2 show their mean and standard deviation. Columns 3–5 present the coefficients, standard error and *p*-value of the variables shown in the first column regressed on the instrument (a count of the number of height-increasing alleles).

	(1) Mean	(2) Std. dev.	(3) Coeff.	(4) Std. err.	(5) *p*-value
**Height** (columns 1 and 2: in cm; columns 3–5: standardized)					
Age 8	132.2	(5.6)	0.430	0.081	<0.001
Age 13	163.3	(7.6)	0.474	0.089	<0.001

**Control variables**					
Age in months at Focus at 8 clinic	103.1	(2.21)	0.018	0.017	0.284
Age in months at Teen Focus 2 clinic	166.0	(2.12)	0.011	0.015	0.466
Age in months at KS3 exam	169.6	(3.76)	−0.033	0.025	0.194
Birth weight (g)	3422	(549)	−1.505	3.699	0.684
Younger siblings under 18 in the household	0.51	(0.65)	−0.003	0.004	0.543
Older siblings under 18 in the household	0.74	(0.74)	0.000	0.005	0.972

Ln(income)	5.32	(0.45)	0.000	0.003	0.995
Father's education	2.43	(1.01)	0.000	0.007	0.986
Mother's education	2.36	(0.88)	0.003	0.006	0.615
Mother's mother's education	1.73	(0.75)	−0.001	0.005	0.829
Mother's father's education	1.84	(0.79)	−0.003	0.005	0.610
Child is not raised by natural father	0.06	(0.23)	0.001	0.002	0.628
Father's social class at the child's birth	3.04	(1.28)	−0.001	0.009	0.894
Mother is employed part-time at 21 months	0.40	(0.49)	0.008	0.003	0.020
Mother is employed full-time at 21 months	0.10	(0.30)	−0.000	0.002	0.906
Father is employed at 21 months	0.92	(0.28)	0.004	0.002	0.038
Index of Multiple Deprivation (IMD)	19.50	(13.95)	−0.014	0.100	0.891

Mother: alcohol in month 1–3 of pregnancy	0.57	(0.50)	0.004	0.003	0.266
Mother: smoked in month 1–3 of pregnancy	0.18	(0.39)	−0.000	0.003	0.917
Breastfeeding	1.88	(1.20)	0.011	0.008	0.207
Mother's age	3.38	(0.91)	0.002	0.006	0.711
Mother's ‘locus of control’	98.88	(9.44)	−0.020	0.065	0.764
Mother's EPDS	6.46	(4.54)	0.025	0.031	0.412
Mother's CCEI	12.88	(7.19)	0.038	0.049	0.443
Teaching score	7.02	(0.93)	0.001	0.007	0.916
Activities (indoor) score	0.69	(0.20)	0.003	0.001	0.032
Activities (outdoor) score	27.89	(4.61)	−0.008	0.032	0.810

*Note*: Rather than height in cm, the analysis uses standardized heights (with mean 100, standard deviation 10).

### 4.5 IV falsification check

Another way to examine the robustness of our IV approach and the validity of our instruments is by undertaking a ‘falsification check’. We do this in two ways. First, we examine the effect of height on an outcome for which we have clear theoretical reasoning that there should *not* be an effect. Second, we examine the effect of height on an outcome for which we have strong beliefs that there *should* be an effect. These approaches, also known in epidemiology as ‘negative control’ and ‘positive control’ methodology respectively, are increasingly adopted in the biomedical field (see e.g. Davey Smith, 2008, Lipsitch et al., 2010). In the first test, we investigate the relationship between children's height and maternal educational level in an OLS and IV analysis. With evidence of a socio-economic gradient in height, we expect a positive association. However, there is no reason to believe there to be a causal effect, and hence, we expect the IV approach to remove this correlation.

Columns 1 and 2 of Table 4 present the results, showing strong positive correlations between maternal education and height in the OLS, which turn insignificant in the IV model. The IV point estimates are sometimes smaller and sometimes larger, with no clear patterns in size or sign of the effects of height measured at different ages. As expected, the standard errors are much larger in the IV, and we cannot reject the null of no effect. The large standard errors however, also preclude us from rejecting the Durbin–Wu–Hausman (DWH) test, suggesting that we cannot distinguish the IV estimates from the OLS estimates.

**Table 4:** Two falsification checks, OLS and IV.

	Maternal education				Weight (measured at the same age as height)			
	**(1)** OLS		**(2)** IV		**(3)** OLS		**(4)** IV	
	Boys	Girls	Boys	Girls	Boys	Girls	Boys	Girls
Height, age 3	0.005⁎⁎⁎	0.005⁎⁎⁎	−0.004	0.012	0.687⁎⁎⁎	0.650⁎⁎⁎	0.738⁎⁎⁎	0.437⁎⁎
	(0.002)	(0.002)	(0.016)	(0.021)	(0.015)	(0.016)	(0.136)	(0.190)
*p*-value DWH test			0.570	0.712			0.706	0.237
No. of observations	2750	2559	2750	2559	2716	2532	2716	2532

Height, age 5	0.005⁎⁎	0.003	0.005	−0.011	0.609⁎⁎⁎	0.595⁎⁎⁎	0.913⁎⁎⁎	0.932
	(0.002)	(0.002)	(0.020)	(0.087)	(0.026)	(0.032)	(0.183)	(0.684)
*p*-value DWH test			0.996	0.864			0.086	0.592
No. of observations	2003	1867	2003	1867	1742	1617	1742	1617

Height, age 8	0.007⁎⁎⁎	0.005⁎⁎	0.001	0.016	0.681⁎⁎⁎	0.698⁎⁎⁎	0.476⁎⁎⁎	0.446⁎⁎
	(0.002)	(0.002)	(0.018)	(0.027)	(0.018)	(0.020)	(0.145)	(0.227)
*p*-value DWH test			0.751	0.674			0.135	0.243
No. of observations	2346	2248	2346	2248	2209	2126	2209	2126

Height, age 11	0.007⁎⁎⁎	0.003	0.005	0.002	0.644⁎⁎⁎	0.640⁎⁎⁎	0.487⁎⁎⁎	0.213
	(0.002)	(0.002)	(0.016)	(0.019)	(0.017)	(0.018)	(0.132)	(0.184)
*p*-value DWH test			0.928	0.988			0.221	0.008
No. of observations	2226	2218	2226	2218	2226	2216	2226	2216

Height, age 13	0.004⁎⁎	0.006⁎⁎	−0.003	0.044	0.604⁎⁎⁎	0.555⁎⁎⁎	0.520⁎⁎⁎	0.392
	(0.002)	(0.003)	(0.016)	(0.029)	(0.016)	(0.024)	(0.155)	(0.245)
*p*-value DWH test			0.641	0.164			0.582	0.492
No. of observations	1925	1942	1925	1942	1925	1940	1925	1940

Height, age 15	0.004⁎	0.008⁎⁎⁎	0.004	0.046	0.633⁎⁎⁎	0.522⁎⁎⁎	0.541⁎⁎⁎	0.860⁎⁎⁎
	(0.002)	(0.003)	(0.018)	(0.032)	(0.023)	(0.029)	(0.166)	(0.289)
*p*-value DWH test			0.991	0.219			0.573	0.223
No. of observations	1631	1758	1631	1758	1630	1753	1630	1753

*Notes*: The estimates come from regressions of (1) maternal education or (2) body weight on height by gender at ages 3, 5, 8, 11, 13 and 15; DWH test=Durbin–Wu–Hausman test (H_0_: OLS is consistent).

⁎ *p*<0.1.

⁎⁎ *p*<0.05.

⁎⁎⁎ *p*<0.01.

In the second falsification check, we examine the effect of height on body weight. As these are highly (positively) correlated, particularly in children who are still growing (e.g. see any children's growth charts), we expect to find strong positive effects. Assuming that height is exogenous to body weight, we also expect the OLS and IV estimates to be similar, though the exogeneity of height in this setting is an assumption.^12^ However, as shown by Tanner (1978) and discussed above, even with severe (prenatal or postnatal) malnutrition, children attain similar heights as their siblings or peers. Hence, assuming that height is exogenous to weight, a substantially different or null IV finding would cast doubt on our IV strategy.

Columns 3 and 4 of Table 4 show strong positive estimates of height on body weight at different ages in both the OLS and IV. A one standard deviation increase in height is associated with a 0.52–0.70 standard deviation increase in weight in the OLS, and a 0.21–0.93 standard deviation increase in weight in the IV. The point estimates are similar in both models, though the standard errors are again much larger in the IV. The Durbin–Wu–Hausman test shows that the majority of the IV estimates are indistinguishable from those estimated by OLS.

Despite the imprecision of the IV approach, the two tests suggest that our instruments perform well. Although this does not guarantee that our IV approach also correctly identifies the causal effect on the other outcomes of interest such as depression or behavior, it does provide support for the argument that both the approach and the instruments are valid to obtain causal estimates of the effects of stature. In Section 5.3, we examine the robustness of these estimates to the use of different combinations of instrumental variables.

## 5 Results

### 5.1 OLS results

We begin by examining the OLS association between height, cognitive skills and mental health. Columns 1 and 2 of Table 5 show a positive association between height, test scores and IQ that halves when controlling for the background characteristics. The actual magnitude of the association is small: controlling for all covariates (the ‘adjusted’ results), a one standard deviation increase in height is associated, for example, with a 0.057 standard deviation increase in girls’ IQ. Comparing this to the effect of within-school-year age on IQ in our data, this corresponds to a difference in test scores between children born approximately one month apart.

**Table 5:** OLS—The unadjusted and adjusted effects of contemporaneous height (ages 8 and 13) on cognitive skills and mental health.

	**(1) Key Stage 3, Age 14**		**(2) IQ test score, Age 8**		**(3) Scholastic self-esteem, Age 8**		**(4) Global self-worth, Age 8**		**(5) Depression, Age 13**	
	Boys	Girls	Boys	Girls	Boys	Girls	Boys	Girls	Boys	Girls
*Unadjusted*										
Height	0.076⁎⁎⁎	0.133⁎⁎⁎	0.121⁎⁎⁎	0.104⁎⁎⁎	0.024	0.036	0.022	0.023	0.003	0.063⁎⁎
	(0.021)	(0.026)	(0.022)	(0.020)	(0.022)	(0.022)	(0.023)	(0.022)	(0.019)	(0.031)

*Adjusted for all covariates*										
Height	0.038⁎⁎	0.086⁎⁎⁎	0.060⁎⁎⁎	0.057⁎⁎⁎	0.005	0.021	0.008	0.019	0.001	0.055⁎
	(0.017)	(0.023)	(0.020)	(0.019)	(0.023)	(0.022)	(0.024)	(0.023)	(0.019)	(0.031)
No. of observations	1559	1590	2300	2222	2153	2117	2153	2117	1896	1932

*Notes*: The estimates come from OLS regressions of the outcome on contemporaneous height by gender; The adjusted analysis includes controls for: birth weight, age in months, number of older and younger siblings, log family income and its square, mother's -, father's -, and mother's parents’ educational level, raised by natural father, social class, maternal age at birth, parents’ employment status, IMD at birth, mother's smoking and drinking during pregnancy, breastfeeding, mother's ‘locus of control’ and mental health (EPDS and CCEI), parental involvement in child development, and their engagement in active activities with their child.

⁎ *p*<0.1.

⁎⁎ *p*<0.05.

⁎⁎⁎ *p*<0.01.

Columns 3–5 examine the relationship between height, the two measures of self-esteem and symptoms of depression. This shows that height is correlated with increases in self-esteem and depression scores, though the estimates are small and generally indistinguishable from the null (the positive association with depression symptoms for girls is the one exception).

Table 6 presents both the unadjusted and adjusted associations between height and behavioral problems. These show that height is unrelated to hyperactivity and conduct problems, but there is a negative correlation with emotional problems. The effects are again small: a one standard deviation increase in height is associated with 0.06–0.07 standard deviations decrease in emotional problems. The results also show a small negative association between height and peer problems for girls.

**Table 6:** OLS—The unadjusted and adjusted effects of contemporaneous height on behavior at age 13.

	**(1) Hyperactivity**		**(2) Emotional problems**		**(3) Conduct problems**		**(4) Peer problems**	
	Boys	Girls	Boys	Girls	Boys	Girls	Boys	Girls
*Unadjusted*								
Height (age 13)	−0.013	−0.005	−0.060⁎⁎⁎	−0.087⁎⁎⁎	−0.002	−0.015	0.011	−0.068⁎⁎
	(0.023)	(0.027)	(0.019)	(0.032)	(0.021)	(0.028)	(0.024)	(0.029)

*Adjusted for all covariates*								
Height (age 13)	−0.001	0.012	−0.059⁎⁎⁎	−0.068⁎⁎	0.004	−0.000	0.009	−0.056⁎
	(0.024)	(0.028)	(0.020)	(0.032)	(0.021)	(0.028)	(0.024)	(0.029)
No. of observations	1647	1668	1644	1670	1644	1670	1643	1669

*Notes*: The estimates come from OLS regressions of the outcome on contemporaneous height by gender; Controls are listed in the note to Table 5.

⁎ *p*<0.1.

⁎⁎ *p*<0.05.

⁎⁎⁎ *p*<0.01.

### 5.2 IV results

Table 7 presents the IV results for cognitive skills and mental health. The unadjusted and adjusted analyses lead to similar conclusions (as expected, since Table 3 showed the instruments to be generally uncorrelated to the covariates). Our instrument predicts height well in all specifications, with a first stage *F*-statistic between 19 and 34 for boys, and 11 and 18 for girls, satisfying Assumption 3.^13^

**Table 7:** IV—The effects of contemporaneous height, instrumented by a count of the number of risk alleles, on cognitive skills and mental health.

	**(1) Key Stage 3, Age 14**		**(2) IQ test score, Age 8**		**(3) Scholastic self-esteem, Age 8**		**(4) Global self-worth, Age 8**		**(5) Depression, Age 13**	
	Boys	Girls	Boys	Girls	Boys	Girls	Boys	Girls	Boys	Girls
*Unadjusted*										
Height	−0.316	0.703⁎⁎	−0.001	0.944⁎⁎	−0.040	−0.148	−0.157	−0.023	0.163	0.237
	(0.203)	(0.298)	(0.203)	(0.367)	(0.210)	(0.301)	(0.212)	(0.309)	(0.162)	(0.323)
*p*-value DWH test	0.031	0.023	0.544	0.002	0.757	0.537	0.393	0.881	0.310	0.587
First stage *F*-statistic	18.99	13.78	26.23	10.90	25.62	10.53	25.62	10.53	24.33	16.94

*Adjusted for all covariates*										
Height	−0.179	0.538⁎⁎	0.102	0.666⁎⁎	−0.017	−0.148	−0.100	−0.072	0.151	0.211
	(0.159)	(0.242)	(0.162)	(0.278)	(0.188)	(0.286)	(0.187)	(0.293)	(0.154)	(0.312)
*p*-value DWH test	0.148	0.030	0.792	0.007	0.907	0.550	0.557	0.755	0.317	0.613
First stage *F*-statistic	19.52	14.36	33.98	12.80	33.64	11.92	33.64	11.92	26.15	18.31

*Notes*: Estimates are obtained from IV regressions of the outcome on contemporaneous height by gender; Controls are listed in the note to Table 5; DWH test=Durbin–Wu–Hausman test (H_0_: OLS is consistent).

^⁎⁎^ *p*<0.05.

Columns 1 and 2 show the IV estimates for KS3 and IQ respectively. These are positive for girls, but indistinguishable from zero for boys. For girls, instrumented height has a large positive effect on both KS3 and IQ, and we reject the Durbin–Wu–Hausman (DWH) test. Despite the much larger standard errors, the IV estimate for girls is larger than the OLS, suggesting that the latter underestimates the true effect. We discuss possible reasons for this below.

Columns 3–5 of Table 7 show that for self-esteem, global self-worth and depression symptoms, the large standard errors mean we cannot reject the null of no effect, though in contrast to the OLS estimates, all three sets of IV coefficients relate increasing height to worse outcomes.

Table 8 presents the IV results for behavioral problems. In contrast to the OLS results in Table 6, the IV estimates in Column 1 of Table 8 show height to be a predictor of hyperactivity in girls. A one standard deviation increase in instrumented height increases the hyperactivity score by about 0.5 standard deviations. Similarly, height appears to be a positive predictor of boys’ emotional problems, with the DWH test rejecting the exogeneity assumption of height. Although not statistically significant, the estimated effect is only slightly smaller for girls’ emotional problems. Finally, columns 3 and 4 show that height increases conduct problems and decreases peer problems for girls, whilst the opposite is found for boys. With large standard errors however, we cannot statistically reject the null of no effect.

**Table 8:** IV—The effects of contemporaneous height, instrumented by a count of the number of risk alleles, on behavior at age 13.

	**(1) Hyperactivity**		**(2) Emotional problems**		**(3) Conduct problems**		**(4) Peer problems**	
	Boys	Girls	Boys	Girls	Boys	Girls	Boys	Girls
*Unadjusted*								
Height (age 13)	0.110	0.492	0.319⁎	0.204	−0.262	0.130	0.334	−0.334
	(0.191)	(0.372)	(0.178)	(0.365)	(0.186)	(0.354)	(0.216)	(0.344)
*p*-value DWH test	0.513	0.133	0.022	0.405	0.139	0.676	0.118	0.417
First stage *F*-statistic	24.89	9.97	24.87	10.31	25.09	10.18	24.58	10.42

*Adjusted for all covariates*								
Height (age 13)	0.093	0.573⁎	0.324⁎⁎	0.207	−0.255	0.170	0.323	−0.182
	(0.171)	(0.337)	(0.163)	(0.336)	(0.165)	(0.323)	(0.198)	(0.309)
*p*-value DWH test	0.576	0.055	0.011	0.396	0.096	0.592	0.096	0.675
First stage *F*-statistic	29.26	12.04	29.37	12.56	29.41	12.38	29.04	12.66

*Notes*: Estimates are obtained from IV regressions of the outcome on contemporaneous height by gender; Controls are listed in the note to Table 5; DWH test=Durbin–Wu–Hausman test (H_0_: OLS is consistent).

⁎ *p*<0.1.

⁎⁎ *p*<0.05.

### 5.3 Instrument specification checks

We investigate the robustness of these results by using several instrument specification checks. First, we re-run the IV analyses using the weighted allele score as the instrumental variable, rather than the simple count of the number of risk alleles. The first as well as second stage results (available from the authors upon request) are very similar to those shown above, suggesting that the imposed structure on the instrument plays less of a role in this application. In fact, if we regress child height on each of the individual SNPs simultaneously, we cannot reject the null that the coefficients are equal to one another.

Second, we specify the nine SNPs as nine instrumental variables, rather than a count of the number of ‘tall’ alleles. As shown in Table 9, Table 10, this leads to a much weaker first stage, reducing the *F*-statistic to between 2 and 4. The point estimates remain similar to those reported above, though they are somewhat closer to zero. One difference is the estimate for girls’ self-esteem. This was negative when using the allele count, but positive when using each SNP separately as an instrument. As we show below, this is probably due to the general imprecision with which these are estimated. The main results, however, are unchanged for both the unadjusted and adjusted regressions: height increases KS3 and IQ for girls (Table 9), and leads to an increase in behavioral problems (Table 10). In addition, the use of nine instruments allows us to test for over-identification using the Hansen *J* test, which we cannot reject in any of the specifications, providing suggestive evidence that the instruments are uncorrelated with the error term.

**Table 9:** IV—The effects of contemporaneous height, instrumented by the nine SNPs simultaneously, on cognitive skills and mental health.

	**(1) Key Stage 3, Age 14**		**(2) IQ test score, Age 8**		**(3) Scholastic self-esteem, Age 8**		**(4) Global self-worth, Age 8**		**(5) Depression, Age 13**	
	Boys	Girls	Boys	Girls	Boys	Girls	Boys	Girls	Boys	Girls
*Unadjusted*										
Height	−0.164	0.358*	0.015	0.419**	−0.057	0.196	−0.072	0.072	0.099	0.123
	(0.167)	(0.184)	(0.185)	(0.182)	(0.189)	(0.206)	(0.190)	(0.202)	(0.139)	(0.228)
*p*-value DWH test	0.122	0.209	0.484	0.080	0.636	0.495	0.616	0.839	0.568	0.915
*p*-value Hansen *J* test	0.514	0.586	0.550	0.177	0.783	0.702	0.511	0.793	0.310	0.748
First stage *F*-statistic	2.90	3.69	3.63	2.96	3.43	2.48	3.43	2.48	3.50	3.61

*Adjusted for all covariates*										
Height	−0.148	0.296*	0.081	0.353**	−0.002	0.161	−0.074	0.058	0.125	0.098
	(0.137)	(0.175)	(0.146)	(0.165)	(0.168)	(0.217)	(0.168)	(0.218)	(0.133)	(0.246)
*p*-value DWH test	0.137	0.139	0.986	0.068	0.946	0.587	0.646	0.912	0.369	0.923
*p*-value Hansen *J* test	0.900	0.554	0.771	0.595	0.619	0.638	0.524	0.739	0.271	0.788
First stage *F*-statistic	3.08	3.02	4.83	2.81	4.55	2.28	4.55	2.28	3.87	3.16

**Table 10:** IV—The effects of contemporaneous height, instrumented by the nine SNPs simultaneously, on behavior at age 13.

	**(1) Hyperactivity**		**(2) Emotional problems**		**(3) Conduct problems**		**(4) Peer problems**	
	Boys	Girls	Boys	Girls	Boys	Girls	Boys	Girls
*Unadjusted*								
Height (age 13)	0.139	0.280	0.199	0.252	−0.143	0.197	0.285	−0.074
	(0.166)	(0.205)	(0.148)	(0.231)	(0.155)	(0.211)	(0.176)	(0.203)
*p*-value DWH test	0.394	0.156	0.058	0.194	0.324	0.274	0.088	0.859
*p*-value Hansen *J* test	0.290	0.345	0.542	0.383	0.158	0.939	0.934	0.468
First stage *F*-statistic	3.61	3.41	3.63	3.46	3.64	3.46	3.60	3.48

*Adjusted for all covariates*								
Height (age 13)	0.148	0.357*	0.206	0.291	−0.119	0.201	0.297*	−0.055
	(0.150)	(0.212)	(0.135)	(0.230)	(0.136)	(0.213)	(0.164)	(0.205)
*p*-value DWH test	0.380	0.082	0.048	0.162	0.316	0.353	0.059	0.923
*p*-value Hansen *J* test	0.283	0.337	0.454	0.312	0.105	0.820	0.965	0.432
First stage *F*-statistic	4.22	3.23	4.25	3.29	4.25	3.29	4.22	3.30

Finally, as discussed in Section 3.2.2, it is possible to examine genetic confounding through pleiotropy (i.e. variants influencing multiple pathways) or LD (i.e. variants being co-inherited) in more detail, using multiple combinations of genetic variants in different IV specifications. We investigate this here by estimating multiple IV models in which – each time – the instrument is defined by a different set of SNPs. We run a different IV regression for all possible sets of instrumental variables, leading to a total of 511 regressions for each outcome.^14^ Obtaining similar estimates with different instrument sets would provide evidence against genetic confounding and increase the confidence in the validity of our findings.

**Fig. 2:**
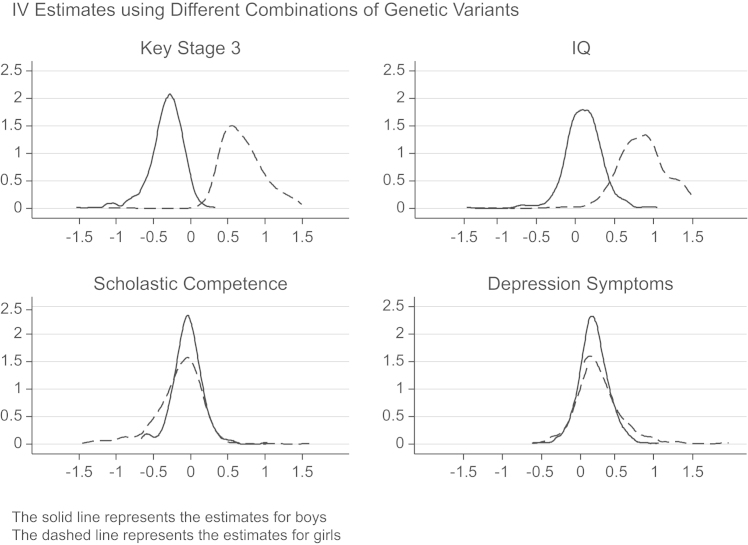
Distribution of point estimates from multiple IV models with different sets of instruments: Cognitive skills and mental health.

**Fig. 3:**
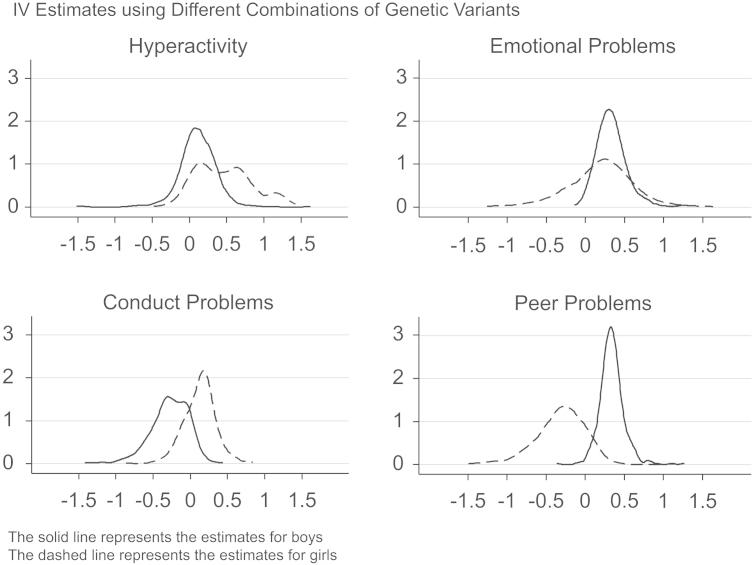
Distribution of point estimates from multiple IV models with different sets of instruments: Behavior.

Fig. 2, Fig. 3 plot the point estimates from the IV regressions with different instrument sets, where the horizontal axis represents the IV estimate.^15^ This shows a clear positive effect of height on KS3 and IQ for girls (the dashed line), with a negative or null effect for boys (the solid line). The sometimes long flat tails of the densities reflect estimates with a first stage *F*-statistic between 1 and 2, for which the estimates are more volatile. Excluding these weaker estimates removes the flat tail. The effect of height on scholastic competence and depression symptoms is generally zero for boys, with girls showing a slightly more positive effect on depression symptoms. In general, the estimates for girls are slightly more variable, which is likely due to their smaller first stage *F*-statistic, which also explains the different findings for self-esteem in Table 7, Table 9. Examining child behavior, the estimates show a clear increase for boys in emotional and peer problems, and a decrease in conduct problems, with no obvious effects on hyperactivity. The effects for girls are slightly more variable, but suggest height increases hyperactivity, emotional and conduct problems, but decreases peer problems.

**Fig. 4:**
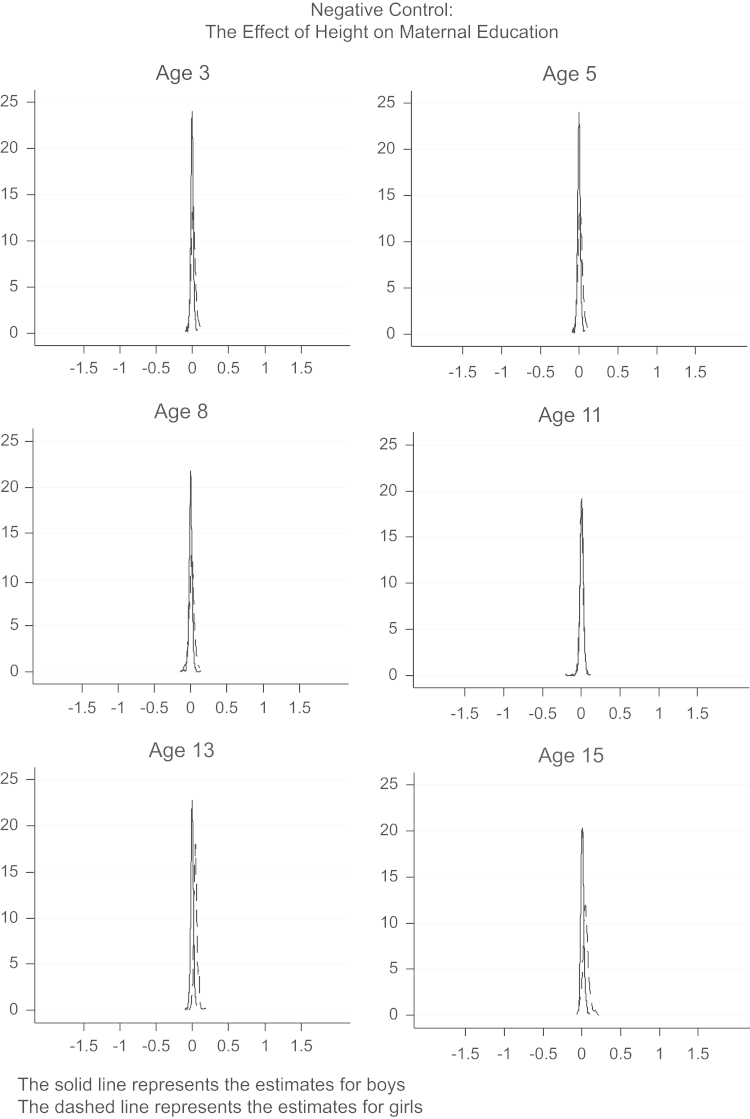
Distribution of point estimates from multiple IV models with different sets of instruments: The effect of height at different ages on maternal education.

**Fig. 5:**
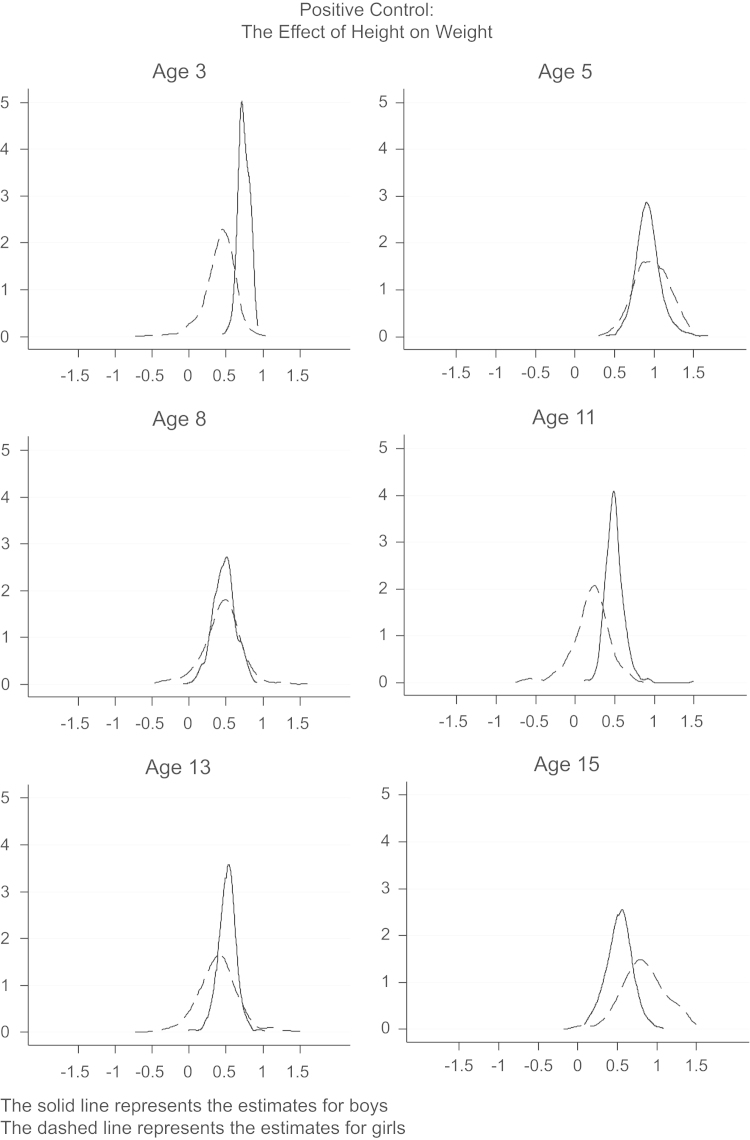
Distribution of point estimates from multiple IV models with different sets of instruments: The effect of height on weight at different ages.

For comparison, Fig. 4, Fig. 5 present the point estimates from IV regressions with different instrument sets for the two falsification checks discussed above and shown in Table 4. The ‘negative control’ clearly shows a spike at zero for both boys and girls, confirming the absence of any effects of height on maternal education. The ‘positive control’ also confirms what we find above: height in both boys and girls increases their weight. Overall, these analyses do not provide evidence against the validity of the IV assumptions.

### 5.4 Non-linearities

As discussed in Section 2, the existing literature has found both tallness and shortness to have negative psychological effects in children. The estimates discussed above only examine differences in the outcome of interest at the mean, but the relationship between height and the outcomes may differ at different points in the distribution. We therefore investigate different cut-points and examine the effects of being below the 25th and above the 75th percentile of the age- and gender specific height distribution. The results (available upon request) confirm our main findings. IV estimates show that shorter girls have lower IQ and do worse in school tests, and vice versa for taller girls, but there is no evidence of a relationship between height and scholastic competence, self-worth or depression. The IV effects of being tall or short on the child's behavioral problems also show similar patterns to those above: relatively tall girls are more hyperactive, and have fewer emotional and peer problems, though with the large standard errors, the latter is not significant at conventional levels. For boys, height increases emotional and peer problems, and decreases conduct problems.

## 6 Discussion and conclusion

This paper is the first to exploit genetic variation in height to examine the causal effects of height on human capital accumulation. OLS results show that taller children perform better in terms of cognitive performance and are less likely to have emotional and peer problems (girls), though tall girls are more likely to show symptoms of depression. Using genetic variation in height in an IV specification, we attempt to deal with the problems of endogeneity. The IV findings for girls are similar to the OLS for cognitive performance, showing a positive effect of height on KS3 and IQ. However, we do not find this for boys, where the results are indistinguishable from zero. We also find no effects of height on self-esteem and depression symptoms. In addition, we find a negative relationship of height with behavior. This suggests that the OLS results are downwardly biased and that height *in*creases rather than *de*creases these behavioral problems. Taller children are more hyperactive and are more likely to have emotional problems. In addition, taller boys are more likely to have peer problems, though there is a negative relationship for girls.

This suggests that height is endogenous to cognitive performance and behavior, though perhaps less so to self-esteem and depression symptoms, for which the OLS and IV estimates do not differ substantively. We are unsure why height would be endogenous to some, but not other outcomes. This may simply be due to the large standard errors, precluding us from making more precise inferences. Alternatively, it may be that unobserved factors such as pre- and postnatal nutrition affect cognitive functioning and behavior, but not self-esteem or depression. We cannot distinguish between such potential explanations.

In many of our results, the IV estimates suggest that OLS is biased downwards. One possible explanation for the difference between IV and OLS could be a genetic one. For example, (one of) our SNPs could be pleiotropic or in LD with another variant that directly affects IQ or cognition. Although our tests of associations with known confounders, our falsification checks, the ‘multiple IV test’, and the scientific literature do not give any reason to expect this to be the case, we cannot rule this out. For instance, it may be that our sample is too small to detect any association between the SNPs and the covariates, and it may be that any pleiotropic effects have simply not yet been identified, or that we do not observe the relevant confounders. From the evidence discussed in Section 3.2 and from the fact that we use only nine SNPs out of possibly hundreds or thousands SNPs coding for height, we assume that our assumptions hold. However, as in any other IV study, we cannot directly test this, and it remains an assumption.

A possible explanation for our IV findings that indicate that being taller *in*creases rather than *de*creases behavioral problems could be the differential treatment of children of different stature. A ‘size-appropriate’ rather than ‘age-appropriate’ treatment of tall children may trigger behavioral problems. Expectations and reactions to ‘tall-for-age’ children's (what may seem childish) behavior can in turn affect children's development. As factors such as socio-economic position are positively related to height and negatively related to behavioral problems, the OLS estimates will be downward biased if these factors are insufficiently controlled for. Though possible, these are speculations as we currently have no further evidence to confirm these. However, the finding of increased behavioral problems is consistent with the psychological literature that has shown a positive relationship between height and children's behavioral problems, though this literature has mainly examined outcomes such as aggression and violence (Raine et al., 1998, Farrington, 1989) rather than those we examine here.

Finally, the IV effects for behavior and IQ are large: a one standard deviation increase in height raises these scores by about 0.2–0.7 standard deviations. Comparing these effects with those of other child characteristics shows they are substantial. For example, a 0.4 standard deviation difference in girls’ IQ (Table 9) is comparable to the difference in this score for girls born approximately 6 months apart within the same school year. Likewise, the difference between girls’ and boys’ raw hyperactivity scores is approximately 0.37 standard deviations which is similar to the estimated effect of one standard deviation increase in height on hyperactivity for girls.

In conclusion, our findings suggest that height is an important factor in children's human capital accumulation in both childhood and adolescence, most likely as a result of the social reactions that are triggered by variations in height. We show that being tall may not only confer advantage but also disadvantage. Our examination of behavioral problems contrasts with the more positive view of height that emerges from the existing empirical literature on height and children's cognitive performance.

## Funding

The UK Medical Research Council (MRC), the Wellcome Trust and the University of Bristol provide core support for ALSPAC. G.D.S. and D.A.L. work in a centre that receives funding from the UK MRC (G0600705) and University of Bristol. Funding from four grants supporting the specific work presented here is gratefully acknowledged: two from the UK Economic and Social Research Council (RES-060-23-0011 and PTA-026-27-2335) and two from the UK MRC (G0601625 and G1002345). No funding body influenced data collection, analysis or its interpretation. This publication is the work of the authors, who will serve as guarantors for the contents of this paper.

## Appendix A brief introduction to genetics

Each cell in the human body contains a nucleus in which most *DNA* (99.9995%) is kept.^16^ DNA provides a template that is ‘read’ and used to produce all of the proteins in living organisms. This is done with just four *bases* (also called nucleotides)—adenine (A), cytosine (C), guanine (G) and thymine (T). DNA consists of two strands of bases, which – for ease of understanding – have been likened to a zip in the way they fasten together. These two strands are held together such that A on one strand can only be linked to T on the other. Similarly, G can only be linked to C. DNA is stored in structures called *chromosomes*, where each chromosome contains a single continuous piece of DNA (made up of the two strands). A *gene* is a section of the chromosome that consists of a segment of DNA, i.e. a set of base pairs in a particular order.

All cells in the human body apart from germ cells contain 46 chromosomes, organized into 23 pairs: one copy of chromosome 1–22 from each parent, plus an X-chromosome from the mother and either an X or Y chromosome from the father.

About 99% of the DNA of any two unrelated individuals is identical. Locations where DNA varies between people are called *polymorphisms*. The most commonly studied form of polymorphism is a *Single-Nucleotide Polymorphism* (SNP): a change in just one base (nucleotide; one of the four molecules that form the codes of DNA) on the DNA sequence. As chromosomes come in pairs (one from each parent), humans have two such bases at each position, called *alleles*. These alleles can either be the same or different. The term *genotype* describes the specific set of alleles inherited at a particular location. For example, individuals can have one of three genotypes of the SNP in *HMGA2* that we use as one of our instruments. They can be:

1. *Homozygous for the common allele* (having two of the same common (most prevalent/typical) alleles. For *HMGA2*, this is denoted by TT)
2. *Heterozygous* (having one common and one rare allele: CT)
3. *Homozygous for the rare allele* (having two of the same rare alleles: CC)

We refer to the height-increasing allele as the ‘tall’ allele. The number of ‘tall’ alleles – and with that an individual's genotype (i.e. being homozygous or heterozygous) – matters, as it is positively related to an individual's height: the more ‘tall’ alleles an individual carries, the taller the individual is on average.

The visible or measurable effect of a particular genotype is the *phenotype* (here: child/adolescent height). Studies of human height generally report a high *heritability*: up to 0.9 (Silventoinen et al., 2003, Macgregor et al., 2006). This however, does not imply that any individual SNP has a large phenotypic effect. For example, there are many different SNPs that affect human height, though all with small effects: so-called ‘polygenes’. Together, these variants may have a large phenotypic effect.

## Footnotes

1 For a brief overview of some of the genetics terms used here, see the glossary in Table 1 and the Appendix.

2 In theory, poor outcomes could lead children to change their eating patterns, which may affect their growth resulting in reverse causation. We argue however, that this is very unlikely and consider this to be less of an issue than (for example) in the case of body weight.

3 In fact, the treatment of short children with growth hormone is, in part, based on the belief that being taller will improve short children's peer relationships (Sandberg et al., 2004, Sandberg and Voss, 2002). However, the evidence showing that short children have more negative experiences is ambiguous: a review by Sandberg and Voss (2002) for example, concludes that the psychological adaptation of shorter-than-average individuals is largely indistinguishable from others, whether in childhood, adolescence or adulthood. Others, however, argue that short people may simply be discriminated against (Magnusson et al., 2006, West, 1991). Persico et al. (2004) instead suggest that taller adolescents are more likely to participate in social activities that develop human capital. Hamermesh and Biddle (1994) argue that (labor market) discrimination does not arise from correlations with height. Instead, they claim it is mainly based on the employee's looks (‘beauty’).

4 See e.g. Weedon et al., 2007, Weedon et al., 2008, Lettre et al. (2008)Gudbjartsson et al. (2008), and Allen et al. (2010). For example, Weedon et al. (2008) identify 20 loci that robustly affect stature, including those used here, using a total of 30,147 individuals of European ancestry. These have since been confirmed in more independent samples (see e.g. Weedon et al., 2007, Lettre et al., 2008, Gudbjartsson et al., 2008, Allen et al., 2010). We use nine of the 20 SNPs identified by Weedon et al. (2008), as these were the only variants available in our data at the time of writing.

5 For a more detailed discussion of the use of genetic markers as instrumental variables from an economic perspective using a similar framework as the above, see von Hinke Kessler Scholder et al. (2011b). Lawlor et al. (2008) includes a more general discussion of the situations and (biological) processes that may invalidate Mendelian randomization studies.

6 Indeed, *DYM*, *GDF5* and *HMGA2* have been associated with skeletal growth in the Online Mendelian Inheritance of Man (OMIM) database (http://www.ncbi.nlm.nih.gov/omim). GDF5 is a protein of the Transforming Growth Factor *β* (TGF*β*) superfamily, which is important for cell growth and skeletal development. *HMGA2* is likely to be associated with increased cell growth, *DYM* is related to skeletal development, and *EFEMP1* and *ADAMTSL3* are involved in pathways influencing the extracellular matrix, which – among others – is essential for processes like growth and wound healing.

7 Even if a genetic component affects both height and the outcome of interest, it does not necessarily imply that a specific genetic variant causes this through (for example) pleiotropy or LD. It may be caused, for example, by interactions between genes (from simple pairs to complex networks), or other variations in DNA.

8 The KS3 scores are averaged over three subjects (English, maths and science) and obtained from the National Pupil Database, a census of all pupils in England within the state school system (this includes 93% of English children), which is matched into ALSPAC. IQ is measured using the Wechsler Intelligence Scale for Children (WISC-III; Wechsler, 1991) and is administered by the ALSPAC psychology team.

9 Family income is an average of two observations (when the child is aged 3 and 4) and is in 1995 prices. The educational indicators are: less than ordinary (O) level, O-level only, advanced (A) level that permits higher educational study, and having a university degree. We use the standard UK classification of social class based on occupation (professional (I), managerial and technical (II), non-manual skilled (IIInm), manual skilled (IIIm), semi-skilled (IV) and unskilled (V)). The Index of Multiple Deprivation (IMD) is based on six deprivation domains, including health deprivation and disability; employment; income; education, skills and training; housing; and geographical barriers to services. Increasing IMD scores indicate greater deprivation. The IMD measure relates to areas containing around 8000 persons.

10 Maternal mental health is measured by the Edinburgh Post-natal Depression Score (EPDS) and Crown-Crisp Experimental Index (CCEI) at 18 weeks gestation. EPDS indicates the extent of post-natal depression; CCEI captures a broader definition of mental health, measuring general anxiety, depression and somaticism. Higher scores mean the mother is more affected. The mother's ‘teaching score’ is constructed from questions that measure whether the mother is involved in teaching her child (depending on the child's age) songs, the alphabet, being polite, etc. We use an average score from three measures at ages 18, 30 and 42 months to capture longer-term involvement. Likewise, a variable is included indicating whether the mother reads/sings to the child, allows the child to build towers/other creations etc., measured at age 24 months. Finally, we account for the extent to which parents engage in active (outdoor) activities with their children, such as going to the park or playground and going swimming.

11 To shed more light on whether the variants are likely to be related to other background characteristics, we also examine the relationship between the genetic variants and a wide set of further variables (64 additional pairwise comparisons) that are not included in our analysis (such as whether the child had sleeping difficulties, the child's ‘locus of control’, whether the mother had a cesarean section, mother's self-esteem, anxiety, depression, whether the family owns their own home, whether they have financial difficulties, etc.). The findings (available from the authors upon request) also suggest the genetic variants are unrelated to these other variables (using a two-sided binomial probability test, *p=*0.77 at the 5% level).

12 If a healthy (unobserved) diet positively affects height and negatively affects weight, the OLS estimates would be biased downwards.

13 As a general test of gene–environment interactions, we explore whether our genetic variants are only expressed in specific environments, and therefore whether there is any direct evidence of violation of the monotonicity assumption. We estimate the first stage regression, interacting the genetic variants with indicators for various subgroups and test whether the instrument coefficient is the same across groups. The results (available from the authors) show no more significant differences than what would be expected by chance, providing suggestive evidence that gene–environment interactions do not play an important role for the genetic variants used here.

14 We generate all possible subsets of *k* SNPs from the total of *n* (nine) elements, where *k*=1,…,9. For example, when using sets of five of the nine SNPs, there are 126 unique combinations (ignoring the ordering of the SNPs): (*n*(*n*−1)(*n*−2)…(*n*−*k*+1))/(*k*(*k*−1)(*k*−2)…1); or (9^⁎^8^⁎^7^⁎^6^⁎^5)/(5!). We repeat this for all *k*-combinations, leading to 511 possible instrument sets.

15 We exclude estimates with a first stage *F*-statistic less than 1. The plots for the two self-esteem measures (scholastic competence and global self-worth) look similar; we plot only the former (the graph for self-worth is available on request).
